# Single nucleus RNA profiling reveals potential therapeutic vulnerabilities in sinonasal carcinomas

**DOI:** 10.1038/s41698-026-01597-6

**Published:** 2026-07-07

**Authors:** Yauheniya Zhdanovich, Christoph Geisenberger, Liliana H. Mochmann, Konstanze Schleich, Edgar Chimal, Fabian Engelhardt-Schott, Linda Bergmayr, Doreen Klingler, Simone Schmid, David Capper, Simon Schallenberg, Frederick Klauschen, Andreas Mock, Philipp Jurmeister

**Affiliations:** 1 Institute of Pathology, Faculty of Medicine, LMU Munich, Munich, Germany; 2 Charité – Universitätsmedizin Berlin, corporate member of Freie Universität Berlin and Humboldt-Universität zu Berlin, Department of Neuropathology, Charitéplatz 1, Berlin, Germany; 3 German Cancer Consortium (DKTK), partner site Berlin a partnership between DKFZ and Charité - Universitätsmedizin, Berlin, Germany; 4 Institute of Pathology, Charité – Universitätsmedizin Berlin corporate member of Freie Universität Berlin and Humboldt-Universität zu Berlin, Berlin, Germany; 5 German Cancer Consortium (DKTK), partner site Munich a partnership between DKFZ and LMU, Munich, Germany

**Keywords:** Cancer, Oncology

## Abstract

Sinonasal undifferentiated carcinomas are rare, aggressive tumors with limited treatment options. Molecular subgroups defined by *IDH2* mutations or SWI/SNF complex deficiencies have recently been recognized, but therapeutic implications remain unclear. We performed single-nucleus RNA sequencing on 12 FFPE tumors (six *IDH2* mutated (IDH2mt), six *SMARCA4* mutated (SMARCA4mt)), generating 59,612 nuclei. Malignant cells were identified by copy number inference, functionally characterized through gene set enrichment analysis, pathway and transcription factor activity analysis, and explored for druggable targets with spatial validation by immunohistochemistry and RNAscope. We identified 17 cell types and four malignant clusters with distinct programs: neuroendocrine-like (enriched in SMARCA4mt tumors), stress-adaptive with ECM remodeling, and EMT/TGF-β-driven. Target expression analysis revealed *KIT* overexpression in IDH2mt tumors and *MET* upregulation in SMARCA4mt tumors. The therapeutic relevance of *KIT* overexpression alone in absence of activating mutations remains uncertain, though treatment with multi-target kinase inhibitors with anti-KIT activity may warrant further investigation. *MET* overexpression may indicate potential relevance of MET-directed antibody-drug conjugate strategies. Both groups showed elevated CDK4 and DDR1 expression, nominating multi-target kinase inhibitors as potential options. Spatial expression uncovered collagenolysis-dependent DDR1 activation in tumor niches, highlighting DDR1 as a promising therapeutic vulnerability. Despite overlapping histology, IDH2mt and SMARCA4mt sinonasal carcinomas exhibit distinct transcriptional states and actionable dependencies. Our findings provide a framework for precision oncology in these rare tumors, supporting evaluation of KIT-, MET-, CDK4/6-, and DDR1-directed approaches.

## Introduction

Sinonasal malignancies are rare tumors that make up around 3% of all head and neck tumors^1^. Despite the small size of this anatomic region, a diverse spectrum of different neoplasms can arise, including poorly characterized tumors such as sinonasal undifferentiated carcinomas (SNUCs). By definition, SNUCs lack specific histomorphological features and have an undifferentiated immunoprofile, aside from potentially limited expression of neuroendocrine markers. The first complete genome and transcriptome analysis of a SMARCB1-negative tumor in the sinonasal region demonstrated the importance of molecular profiling for the diagnosis of rare cancer types^2^. Previous studies identified recurrent molecular alterations such as *IDH2* mutations or SMARCB1-deficiency (either caused by biallelic loss or inactivating mutations of *SMARCB1*)^3,4^. Based on these findings, the definition of SNUCs has been updated in the recently revised World Health Classification (WHO) of Head and Neck Tumors. While undifferentiated carcinomas with *IDH2* mutations are still referred to as SNUCs, a new entity named SWI/SNF complex-deficient sinonasal carcinoma has been introduced. These tumors also harbor an undifferentiated phenotype but are molecularly defined by SMARCB1-deficiency or *SMARCA4* loss of function mutations. DNA methylation-based clustering analysis demonstrated that *IDH2* mutated carcinomas form a compact and distinct epigenetic cluster clearly separated from SMARCB1-deficient carcinomas. In addition, patients with *IDH2* mutated SNUCs seem to have a more favorable prognosis compared to other undifferentiated subtypes^5–7^.

Despite these early advances in tumor classification, there is limited knowledge about biology-informed treatment options for these patients. SNUCs with *IDH2* mutations have a molecular rationale for treatment with anti-IDH2 agents, however drug efficacy could not be sufficiently shown thus far^8^. SWI/SNF complex deficient carcinomas might be sensitive to EZH2 inhibitors, but the available clinical data is limited to a small case number of patients with mixed results^8,9^. Due to the overall limited clinical data and lack of model systems, further biology- and data-driven insights are warranted to identify novel putative treatment targets.

Recently, single nucleus RNA sequencing (snRNA-seq) protocols have been optimized to allow the analysis of formalin-fixed and paraffin-embedded (FFPE) tissue samples^10–13^. Early studies using FFPE-derived material for snRNA-seq are comparable to fresh tissue and may have overall superior quality to frozen specimens^14^. This opens up new possibilities for in depth-profiling of rare tumor entities as samples can easily be retrieved from clinically annotated pathology archives without the need for prospective tissue collection. In order to gain novel insights into this poorly characterized and exceedingly rare tumor entity, we performed snRNA-seq on archived sinonasal carcinomas with undifferentiated morphology. Building on our recent DNA methylation-based study, we focused on two molecular subtypes proposed in that work, which we had provisionally designated as “NEC-like IDH2” and “NEC-like SMARCA4/ARID1A”^6^. Although neither group constitutes a defined tumor entity in the current WHO classification, both show overlapping histomorphological features and evidence of neuroectodermal differentiation, while exhibiting clearly distinct epigenetic profiles and mutational landscapes^6^. To further investigate their molecular background, we selected a cohort of tumors harboring *IDH2* mutations or *SMARCA4* mutations for further investigation.

**Fig. 1:**
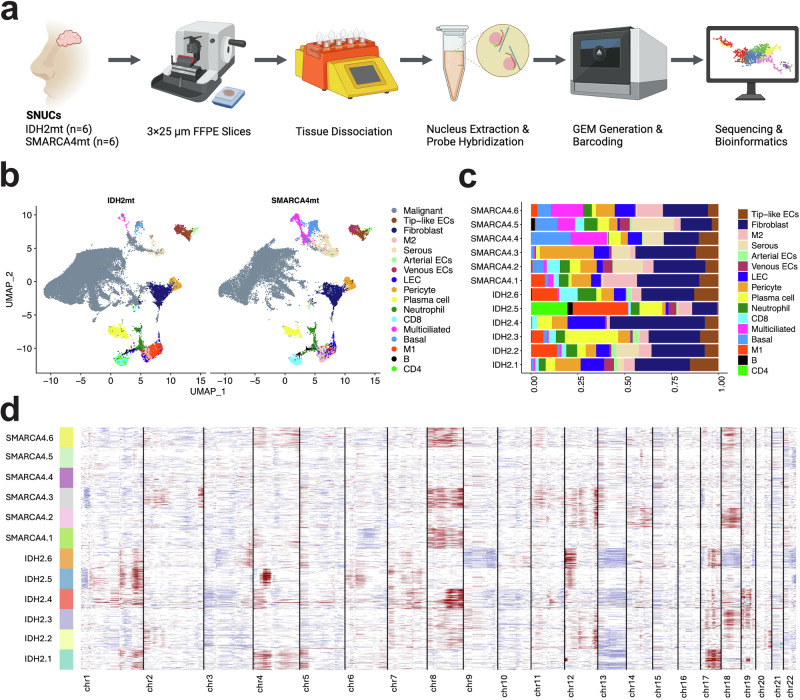
Single nucleus RNA sequencing (snRNA-seq) of IDH2mt and SMARCA4mt sinonasal carcinomas. **a** Cohort overview and schematic workflow display the sample preparation for snRNA-seq. **b** UMAP visualization of the filtered cohort of 59,612 cells stratified by mutation status (*IDH2* mutated (IDH2mt), *SMARCA4* mutated (SMARCA4mt)) and colored by cell cluster. IDH2mt and SMARCA4mt snRNA-seq profiles showed a joined single-cell landscape with prevalence of all major stromal and immune cell types. **c** Relative RNA abundance of non-malignant cells to the total non-malignant population for each of the 12 samples is plotted. **d** A heatmap of large-scale copy number variations for individual tumor cell nuclei (rows) is inferred from snRNA-seq data. For the visualization, each sample was downsampled to the size of the smallest sample (338 malignant cells).

Our results show that *IDH2* mutated (IDH2mt) and *SMARCA4* mutated (SMARCA4mt) sinonasal carcinomas exhibit distinct cellular landscapes, transcriptional profiles, and potential therapeutic targets. We generated snRNA-seq profiles of 12 samples, including six IDH2mt and six SMARCA4mt samples. A graphical representation of the study design is shown in Fig. 1a. We identified 17 cell types and observed that IDH2mt and SMARCA4mt tumors share a broadly similar single-nuclei landscape enriched with all major cell types (Fig. 1b, c). We defined four malignant clusters (MC1-MC4) with distinct functional roles. Therapeutic target analysis revealed that IDH2mt tumors showed high *KIT* expression and SMARCA4mt tumors elevated *MET* expression, pointing to receptor tyrosine kinase programs with potential therapeutic relevance. In the absence of activating mutations, these findings could inform the future evaluation of treatment options involving e.g., multi-target kinase inhibitors with anti-KIT activity and MET-directed antibody-drug conjugate strategies. Both groups showed *CDK4* and *DDR1* expression, identifying multi-target kinase inhibitors dasatinib and imatinib as additional potential therapeutic options. Our findings provide a comprehensive framework for understanding tumor heterogeneity and offer a data-driven approach to identifying therapeutic opportunities in IDH2mt and SMARCA4mt sinonasal carcinomas.

## Results

### Single-cell landscape of IDH2 and SMARCA4 mutant sinonasal carcinomas

To explore the cellular compositions and oncogenic dependencies in sinonasal carcinomas we generated snRNA-seq data for six IDH2mt and six SMARCA4mt primary tumors. On average 5558 nuclei (range 1328–11,623) could be sequenced per sample with 11,081 median reads per nucleus (range 4514–21,968) and 78.58% confidently mapped reads (range 62.62–93.95%; Suppl. Table S1). There was no significant correlation between the age of FFPE blocks and each of the quality metrics: (1) nuclei count, (2) number of median reads per nucleus, and (3) percentage of confidently mapped reads (Spearman correlation, *p* > 0.05).

We retained 66,690 nuclei from the 12 patients with 59.06% and 40.94% nuclei originating from IDH2mt and SMARCA4mt tumors, respectively. A total of 7078 nuclei were filtered according to the quality control criteria, including sample-specific thresholds for gene count, mitochondrial gene expression and doublets detection (see Methods). After filtering, the final dataset comprised 59,612 nuclei. Malignant cells were distinguished from non-malignant cells by inference of large-scale chromosomal CNVs based on binned expression levels within the nuclei transcriptomes (Fig. 1d). The classes of non-malignant cells were identified using a combination of multimodal reference mapping of ten published RNA seq datasets and RNA expression of cell-type-defining marker genes (Suppl. Fig. S1). IDH2mt and SMARCA4mt samples showed a joined single-cell landscape with prevalence of all major non-malignant cell groups mostly in all samples (Fig. 1b, c). The inferred CNVs clustered nuclei by sample of origin and were consistent with the copy number changes determined by DNA methylation microarray analysis of the same samples (Suppl. Fig. S2). In addition, stratification of inferred CNV profiles by malignant clusters (MC1-MC4) did not reveal cluster-specific copy number patterns. Large-scale chromosomal alterations were largely shared across clusters and primarily reflected the sample of origin rather than transcriptional state (Suppl. Fig. S3).

We annotated 17 cell types based on RNA expression levels as malignant cells, tip-like endothelial cells (ECs), arterial ECs, venous ECs, lymphatic endothelial cells (LEC), fibroblasts, pericytes, B cells, CD4 and CD8 T cells, plasma cells, multiciliated cells, basal cells, serous cells, neutrophils, M1 and M2 macrophages.

### Analysis of intratumoral heterogeneity reveals different functional tumor states

**Fig. 2:**
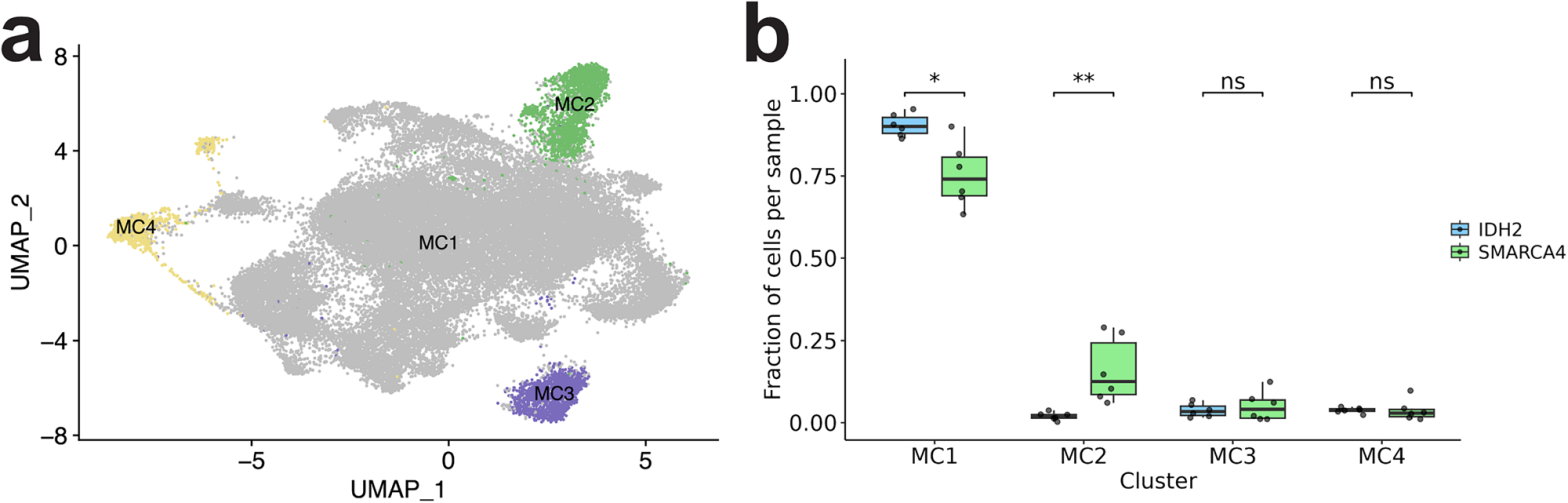
An analysis of tumor cell heterogeneity in IDH2 and SMARCA4 mutants. **a** A UMAP clustering of the four malignant cell clusters MC1-MC4 is displayed. **b** The fraction of malignant cells assigned to each cluster (MC1-MC4) is plotted. The jittered data points of boxplots represent fraction values for individual IDH2mt and SMARCA4mt samples. The fraction was computed as the number of cells assigned to a cluster in a sample divided by the total number of malignant cells of the same sample. MC2 is predominantly present in SMARCA4mt samples, *p* < 0.01.

Next, we focused on tumor cells across samples (total of 40,390 tumor cells with median of 3,429 genes per cell) by extracting only the epithelial population with copy number alterations (Fig. 1d). After isolating all tumor cells, we identified four distinct malignant cell clusters MC1-MC4 (Fig. 2a). MC1, MC2, MC3, and MC4 contained 35,343, 2172, 1543, and 1332 cells respectively. While MC1, MC3, and MC4 contained nuclei from both SMARCA4mt and IDH2mt samples, MC2 mainly contained nuclei from SMARCA4mt samples (*p* < 0.01, Wilcoxon-Mann-Whitney test, BH method; Fig. 2b).

### Functional characterization

**Fig. 3:**
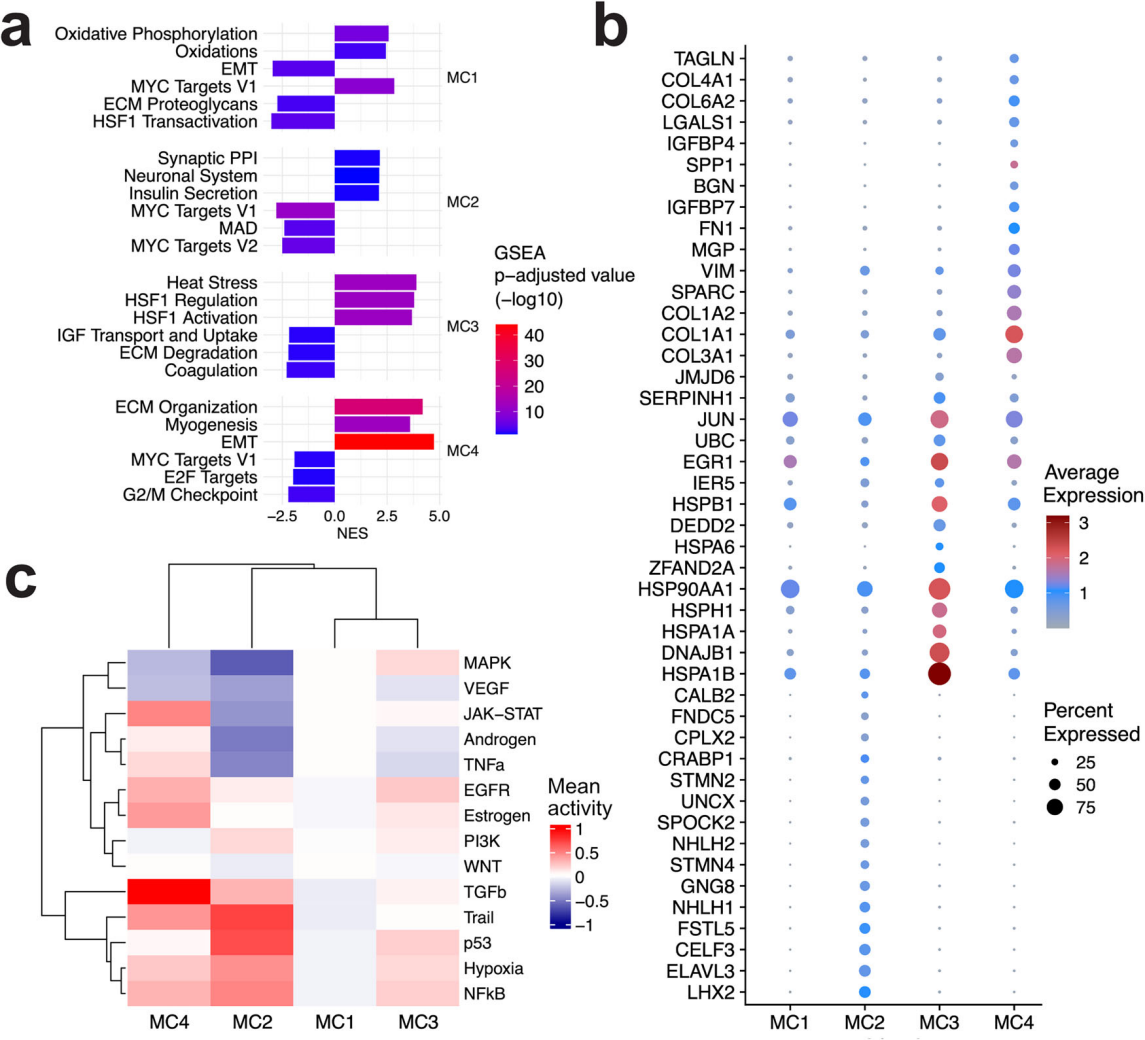
Functional characterization of tumor clusters. **a** Gene set enrichment analysis (GSEA) reveals distinct biological processes associated with malignant clusters (MC1-MC4), including metabolic processes and immune functions in MC1, nervous system development in MC2, cellular responses to stimuli in MC3, and epithelial-mesenchymal transition (EMT) in MC4. **b** Expression levels (color intensity) and percentage of cells (dot size) expressing differential gene markers for MC1-MC4. **c** Pathway activity analysis highlights differences in pathway enrichment across MC1-MC4. MC1 shows no significant pathway activity. MC2 shows the highest activity in pathways Trail and p53, while MC3 exhibits increased p53, hypoxia, and NFkB pathway activities. TGF-β is mostly activated in MC4 suggesting its involvement in EMT.

Gene set enrichment analysis demonstrated that MC1 was enriched for pathways associated with enhanced metabolic activity, epithelial-mesenchymal transition (EMT), transcriptional activation by MYC, and heat shock factors (Fig. 3a). For MC2, a neuronal/neuroectodermal identity with active mitochondrial and metabolic regulation is enriched in pathways related to the neuronal system (e.g., protein-protein interactions at synapses, neuronal system), mitochondrial protein degradation, and MYC targets. MC3 indicated high enrichment of cellular stress responses (e.g., cellular response to heat stress, HSF1-mediated heat shock response) and extracellular matrix (ECM) degradation. MC4 reflected strong cell-cycle progression (E2F, MYC targets, G2/M checkpoint) and mesenchymal traits with enrichment in extracellular matrix organization, myogenesis, and EMT pathway, a hallmark of cancer progression and metastasis^15,16^.

Figure 3b contains differentially expressed genes determined for each MC. MC1 did not show any DEGs that were exceptionally specific to this cluster only. Consistent with the GSEA findings, MC2 is characterized by neuronal/neuroectodermal identity and active neural differentiation, supported by DEGs such as *LHX2*, *NHLH1*, and *UNCX*, which regulate neuronal development^17,18^. Additionally, the involvement of *STMN2*, *STMN4* in neuronal growth through microtubule dynamics, and *CALB2* as a modulator of neuronal excitability further underscores the cluster’s specialized neuronal/neuroectodermal profile^18^. Multiple heat shock proteins (including *HSPA1B*, *HSPA1A*, *HSPH1*, *HSP90AA1*, and *HSPB1*) were identified among the DEGs in MC3, further validating the findings from the GSEA. Among DEGs for MC4 were multiple collagens from the collagen family which are expressed in the ECM^19^. *SPARC* and *FN1* are involved in cell-matrix interactions, influencing cell adhesion, proliferation, and migration^18^.

Pathway activity analysis further supported the EMT phenotype of MC4 tumor cells (Fig. 3c). Strong activation of the transforming growth factor-β (TGF-β) pathway indicated that MC4 was likely involved in EMT, tumor invasion, and metastasis^20^. MC1 showed no significant pathway activity. In MC2, TRAIL and p53 activation indicated a focus on apoptosis and tumor suppression mechanisms. p53 activation in MC3 suggested some tumor suppressive signaling, though less prominent compared to MC2.

In light of a recent study identifying ASCL1, NEUROD1, POU2F3, YAP1 as novel neuroendocrine markers in sinonasal tumors^21^, we examined their activation in our cohort.

While *ASCL1, NEUROD1*, and *YAP1* expression remained low across all malignant clusters and samples, elevated *POU2F3* expression was observed predominantly in IDH2mt tumors and clusters MC1 and MC4 (Suppl. Fig. S4A, D). POU2F3 program scores were significantly higher in IDH2mt tumors compared with SMARCA4mt tumors (Wilcoxon-Mann-Whitney test, *p* < 0.005), indicating activation of a tuft cell-associated transcriptional program (Suppl. Fig. S4C, F).

In contrast, neuroendocrine (NE) differentiation scores were generally low in IDH2mt tumors and in clusters MC1, MC3, and MC4, with relative enrichment confined to MC2 and predominantly observed in SMARCA4mt tumors (Suppl. Fig. S4B, E). These findings are consistent with the previous identification of neuroendocrine-like activity of MC2 and its enrichment in SMARCA4mt tumors.

### Transcriptional metaprograms define distinct malignant cell states and their regulatory programs

To systematically characterize transcriptional heterogeneity across malignant tumor cells, we applied non-negative matrix factorization (NMF) and identified ten consensus gene expression metaprograms (MPs). Five MPs were excluded from downstream analysis due to limited robustness (Suppl. Fig. S5A). Functional annotation of the remaining metaprograms was performed using gene set enrichment analysis complemented by manual curation, allowing classification of biologically coherent transcriptional programs (Suppl. Fig. S5B). Among these, MP8 was defined by strong enrichment of neuronal differentiation and neurodevelopment-associated gene sets. In line with our previous results, MP8 activity was highest in malignant cluster MC2 (Suppl. Fig. S5C), which we previously classified as neuroendocrine-like. Moreover, MP8 activity was significantly enriched in SMARCA4-altered tumors (Suppl. Fig. S5D), reinforcing an association between this genetic background and neuronal-like differentiation programs. These findings indicate that malignant clusters are driven by distinct transcriptional programs reflecting divergent differentiation states.

To further dissect the regulatory mechanisms, we additionally estimated transcription factor activity across MC1-MC4 (Suppl. Fig. S6A). MC1 exhibited no detectable TF activity, consistent with its lack of significant pathway activation. MC2 showed high activity of *ATOH7*, *NEUROG3*, *PHOX2A*, *BCL11A*, and *EBF1*, aligning with its enrichment in nervous system development and neurogenesis-associated pathways^22^. MC3 was characterized by elevated activity of *NFYB*, *NFYC*, *HSF1*, and *HSF2*, supporting its role in cellular stress responses and adaptation to external or internal signals, as indicated by its enrichment in cellular response pathways and p53 activation. Finally, MC4 demonstrated strong activity of *SCX*, *RUNX2*, *ZBTB7B*, *SMAD3*, and *TCF21*, supporting its enrichment in EMT pathways and TGF-β activity^23^. This highlighted the involvement of MC4 in tumor invasion and metastasis.

### Evaluation of therapeutic kinase targets and immunoregulatory interactions

**Fig. 4:**
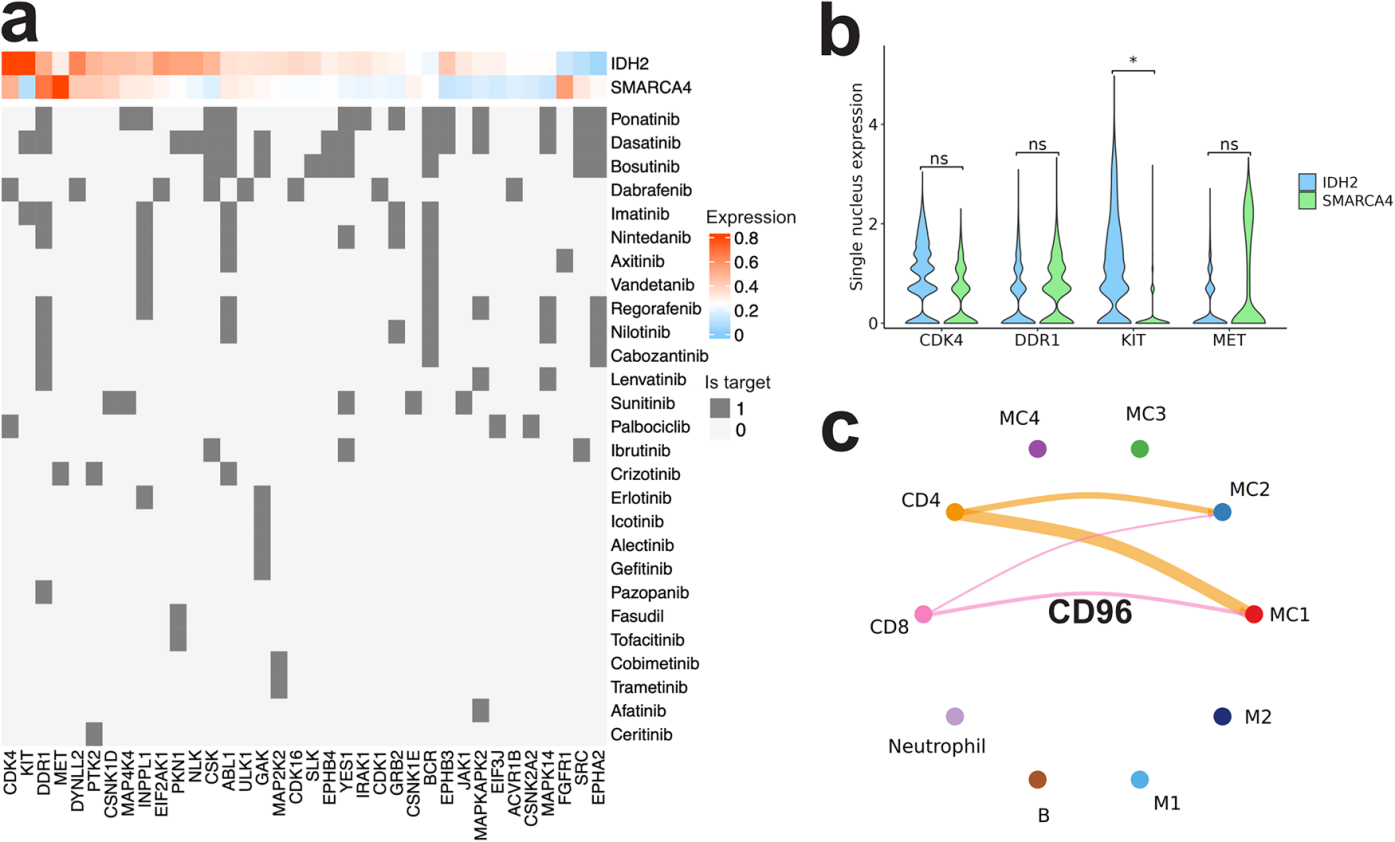
Therapeutic target landscape and pathway analysis of *IDH2* mutated (IDH2mt) and *SMARCA4* mutated (SMARCA4mt) groups. **a** Heatmap shows the expression of clinical kinase drug targets and their corresponding inhibitors in IDH2mt and SMARCA4mt groups. Key findings include *KIT* overexpression in the IDH2mt and *MET* overexpression in the SMARCA4mt group. **b** Violinplots show the gene expression of *CDK4*, *DDR1*, *KIT*, and *MET* single nuclei, stratified by tumor subtype (IDH2mt in blue, SMARCA4mt in green). *KIT* showed significantly higher expression in IDH2mt tumors (*p* < 0.05; Wilcox-test on pseudobulk), whereas *CDK4* and *DDR1* did not show significant differences between groups. Points represent individual nuclei and significance levels are indicated above each gene. **c** CD96-mediated cell-cell communication. CD4 and CD8 T cells exhibit strong outgoing interactions toward malignant clusters MC1 and MC2. Edge thickness reflects communication probability inferred by CellChat.

Exploration of the target landscape of clinically available tyrosine kinase inhibitors within the SMARCA4mt and IDH2mt groups revealed distinct expression patterns that could guide potential therapeutic strategies (Fig. 4A). In the IDH2mt group, we identified high expression of *KIT* (Fig. 4B), highlighting the potential efficacy of KIT inhibitors such as dasatinib and imatinib as therapeutic options. In contrast, the SMARCA4mt group showed high expression of *MET*, suggesting crizotinib or MET-directed antibody drug conjugates (ADC) as a targeted therapeutic candidate for SMARCA4mt tumors.

Additionally, *CDK4* was highly expressed in both IDH2mt and SMARCA4mt groups, with higher expression levels in IDH2mt samples (Fig. 4b). This suggests that inhibitors such as dabrafenib and palbociclib could be effective in both groups, with a potentially stronger effect in IDH2mt tumors.

*DDR1* was expressed in both groups, with an even more pronounced expression in SMARCA4mt samples (Fig. 4b). This indicates that DDR1 inhibitors, including multiple compounds such as dasatinib or imatinib, could be particularly relevant for SMARCA4mt tumors. Importantly, dasatinib and imatinib represent particularly promising candidates due to their broad targeting capability, as they could inhibit KIT in IDH2mt tumors and DDR1 in both tumor types.

Supplementary Fig. S6B further highlights the expression patterns of clinical kinase drug targets across MC1-MC4, complementing our observations in the IDH2mt and SMARCA4mt groups. CDK4 and DDR1 were broadly expressed across all clusters. MC1, MC3, and MC4 showed high KIT expression reflecting the predominance of IDH2mt cells.

Cell-cell communication analysis identified CD96-mediated interactions between CD4 and CD8 T cells and the malignant clusters MC1 and MC2 (Fig. 4c). These interactions were among the strongest inferred signaling events and were consistently observed across both IDH2mt and SMARCA4mt tumors.

### Orthogonal validation of key molecular alterations

To determine whether the findings identified by snRNA-seq were supported by clinically established assays, we performed orthogonal validation using targeted DNA sequencing, immunohistochemistry, copy number profiling, and fluorescence in situ hybridization (FISH).

In addition to the snRNA-seq analyses, we performed targeted DNA sequencing using a suitable, targeted gene panel on all *IDH2* and *SMARCA4*-mutated samples. These analyses confirmed the respective driver alterations and revealed few additional pathogenic mutations, most notably in *TP53* (2 cases), *CTNNB1* (2 cases) and *PIK3CA* (2 cases; Suppl. Table S2). Importantly, no activating *KIT* mutations were identified in any of the analyzed cases, including tumors with high *KIT* expression. Furthermore, no *MET* exon 14 skipping alterations were identified in the SMARCA4mt samples.

We performed immunohistochemical analyses for KIT and MET on all samples. Quantitative assessment using an H-score approach demonstrated a strong correlation between mRNA expression levels derived from snRNA-seq and protein expression levels by immunohistochemistry (Spearman correlation, p_KIT < 0.01, ρ_KIT = 0.7474; p_MET < 0.001, ρ_MET = 0.8732; Suppl. Fig. S7A). Representative hematoxylin and eosin and immunohistochemical stainings further illustrated concordant high and low expression patterns in IDH2.1 and SMARCA4.4 tumors (Suppl. Fig. S7B).

*MET* amplification was evaluated by FISH on FFPE sections (Suppl. Table S3) and by copy number profiling derived from DNA methylation arrays (Suppl. Fig. S8). Neither method demonstrated evidence of *MET* amplification, indicating that *MET* overexpression is not driven by genomic amplification.

In addition, we performed histopathologic validation of SMARCA4mt tumors using conventional hematoxylin and eosin staining and BRG1 immunohistochemistry (Suppl. Fig. S9). Tumors harboring truncating *SMARCA4* mutations demonstrated complete loss of BRG1 expression. In contrast, cases with missense mutations retained BRG1 protein expression, likely because the antibody epitope remains preserved.

### Spatial validation of activation of the collagen-DDR1 signaling axis

Given the notable overlap in therapeutic kinase targets in IDH2mt tumors, particularly DDR1 and KIT, we set out to further elucidate the DDR1 signaling pathway as a potential therapeutic vulnerability in these sinonasal carcinoma subtypes. Recent work identified a collagenolysis-dependent signaling axis in pancreatic cancer, wherein matrix metalloproteinase-cleaved collagen I activates DDR1, which subsequently drives NFkB-mediated expression of p62 (SQSTM1) and NRF2, ultimately enhancing mitochondrial biogenesis and tumor metabolism^24^. Conversely, intact collagen I promotes proteasomal degradation of DDR1, thus suppressing this oncogenic signaling pathway.

**Fig. 5:**
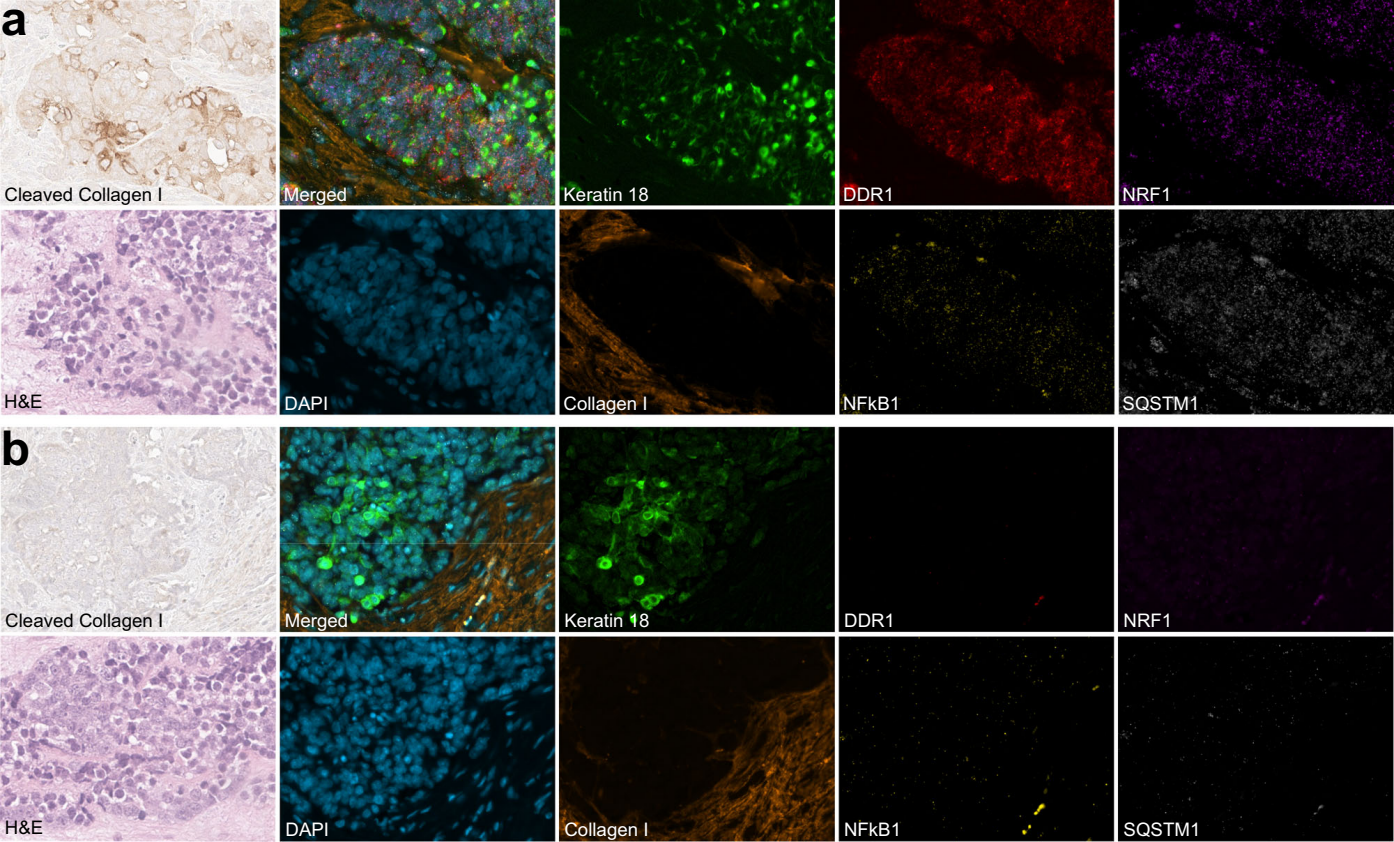
Multimodal comparison of cleaved collagen I-positive and -negative areas of an IDH2mt tumor using conventional immunohistochemistry, sequential immunofluorescence (seqIF) and RNAscope analysis. **a** Representative region demonstrating clear membranous immunohistochemical staining for cleaved collagen I on the surface of tumor cells. seqIF highlights tumor cells and collagen-rich stroma specifically with antibodies against Keratin 18 and Collagen I, respectively. RNAscope analysis utilizing probes targeting DDR1, NRF1, NFkB1, and SQSTM1 reveals strong expression in tumor cells, whereas adjacent fibroblasts show negligible signal. **b** Representative region lacking immunohistochemical positivity for cleaved collagen I. Consistent with our hypothesis, DDR1 and its downstream effectors exhibit minimal to no expression in the corresponding RNAscope analysis.

To determine the spatial activation of this pathway in sinonasal carcinomas, we established an immunohistochemical (IHC) assay with an antibody specifically targeting the collagen I cleavage site, accompanied by a sequential immunofluorescence (seqIF) panel (CK18, Collagen I) and RNAscope probes for DDR1 and corresponding downstream targets (NRF1, NFkB1, SQSTM1). Our analysis clearly showed significant enrichment of DDR1 signaling and its downstream effectors in tumor regions positive for cleaved collagen I (Fig. 5a) compared to regions without cleaved collagen I (Fig. 5b; Suppl. Table S4), underscoring the activation of this pathway in collagen-remodeled tumor niches. These findings substantiate the therapeutic potential of targeting the DDR1-collagen signaling axis in sinonasal carcinomas.

## Discussion

Sinonasal carcinomas remain among the most challenging tumors of the head and neck region, characterized by aggressive clinical behavior, limited treatment options, and historically vague histopathological definitions. Recent WHO classifications have refined their taxonomy by recognizing molecularly defined subsets such as IDH2mt sinonasal undifferentiated carcinoma and SWI/SNF-deficient sinonasal carcinoma, yet the therapeutic consequences of these distinctions remain uncertain. In this study, we advance the understanding of these rare entities by providing the first single-nucleus RNA atlas of IDH2mt and SMARCA4mt sinonasal carcinomas. Beyond confirming subtype-specific molecular hallmarks, our data identify distinct transcriptional programs and druggable dependencies, thereby bridging molecular classification with potential therapeutic strategies.

The analysis of malignant subclusters revealed that sinonasal carcinomas are not homogeneous, but instead consist of divergent cellular states with unique functional attributes. Among these, a distinct cluster enriched in SMARCA4mt tumors exhibited transcriptional programs reminiscent of neuronal/neuroectodermal differentiation, which is consistent with prior reports documenting expression of neuroendocrine and neuronal markers such as NSE, synaptophysin, and UCHL1, as well as the recurrent observation of rosette-like structures^6^.

To systematically characterize malignant cell heterogeneity, we applied non-negative matrix factorization and identified robust transcriptional metaprograms defining distinct cellular states. MP8 was enriched for neuronal differentiation and showed highest activity in MC2, with significant enrichment in SMARCA4mt tumors, reinforcing their neuroendocrine-like phenotype. Consistent with recent reports on lineage-defining transcription factors in sinonasal tumors, neuroendocrine differentiation scores were preferentially enriched in this cluster, whereas *ASCL1*, *NEUROD1*, and *YAP1* remained largely inactive^21^.

In contrast, *POU2F3* expression and program scores were significantly elevated in IDH2mt tumors, particularly within MC1 and MC4, indicating activation of a tuft cell-associated transcriptional program. Transcription factor activity analysis further supported these lineage distinctions, with neurodevelopment-associated regulators enriched in the neuronal-like cluster and TGF-β/EMT-related factors predominant in MC4. Together, these findings suggest that IDH2mt and SMARCA4mt sinonasal carcinomas occupy distinct lineage states rather than representing variants along a shared differentiation spectrum.

Another cluster was characterized by strong expression of stress-response and heat-shock pathways, underscoring the role of adaptive survival mechanisms in aggressive tumor phenotypes. Inhibitors of HSP90 and other chaperones, which have demonstrated efficacy in other cancers dependent on stress adaptation, may represent one avenue of intervention^25,26^. By contrast, a distinct subset of cells displayed pronounced TGF-β and EMT signatures, aligning with biological processes known to drive invasion, metastasis, and immune evasion in other related tumors such as head and neck squamous carcinomas^27^. The TGF-β pathway plays a dual role in cancer, acting as a tumor suppressor in early stages by inhibiting cell proliferation and promoting apoptosis, but transitioning to a pro-tumorigenic role in advanced stages by inducing EMT, enhancing cell motility and fostering immune evasion^28^.

The coexistence of neuroendocrine-like, stress-adapted, and EMT-driven states within single tumors highlights the profound intratumoral heterogeneity of sinonasal carcinomas and provides a rationale for the frequent therapeutic resistance observed in clinical management of SNUC patients.

The therapeutic target analysis provides additional context that complements these biological observations. In our cohort, *KIT* expression was high and largely restricted to IDH2mt tumors. KIT is a clinically actionable kinase, with imatinib and related inhibitors representing a precedent for targeted therapy in KIT-dependent tumors such as gastrointestinal stromal tumors^29^. Notably, the only response to imatinib in SNUC reported in the literature involved a *KIT*-mutant case^30^. Therefore, it remains uncertain whether high *KIT* expression alone, in the absence of an activating mutation, confers comparable sensitivity. The potential of IDH2 as a target is currently being investigated in a Phase II trial of Enasidenib for IDH2mt SNUC and skull base tumors^31,32^.

In SMARCA4mt tumors, the identification of *MET* upregulation points toward potential benefit from MET-targeted antibody-drug conjugates such as telisotuzumab vedotin, already established in other MET-dependent solid tumors such as non-small cell lung cancer^33,34^.

More broadly, we emphasize that kinase overexpression should be interpreted as a hypothesis-generating therapeutic vulnerability rather than definitive evidence of drug sensitivity. Nevertheless, expression-based nomination of kinase targets is supported by precedent across tumor types, including *FGFR* mRNA-guided rogaratinib treatment, *EGFR* mRNA-associated gefitinib sensitivity, and receptor tyrosine kinase expression-associated response to sunitinib^35–37^. Thus, while *KIT* and *MET* expression alone do not establish sensitivity to imatinib, dasatinib, or crizotinib, these findings may support future functional and translational studies of kinase-directed strategies in these rare tumors.

Across both groups, *CDK4* was consistently expressed, suggesting a potential role of CDK4/6 inhibitors that are already widely used in other malignancies and have not yet been evaluated for sinonasal tumors. For instance, CDK4/6 inhibitors have received clinical approval for hormone receptor (HR)-positive/human epidermal growth factor receptor 2 (HER2)-negative breast cancer^38,39^. A recent trial in advanced sarcoma evaluated palbociclib efficacy in patients selected based on *CDK4/CDKN2A* mRNA expression, illustrating that kinase expression may help nominate therapeutic vulnerabilities for further evaluation^40^. Furthermore, preclinical studies indicate that selective inhibition of CDK4 may enhance therapeutic outcomes by mitigating the toxicity associated with dual CDK4/6 inhibitors^41^.

Perhaps the most unique and intriguing vulnerability lies in the collagen-DDR1 signaling axis. DDR1 has been increasingly recognized as a critical mediator of extracellular matrix remodeling, driving collagen reorganization, fostering immune exclusion and activating immunosuppressive pathways^42,43^. Recent pan-cancer studies reported high expression of DDR1 across multiple tumor entities and association with immunotherapy resistance, poor clinical outcome, and reduced antitumor immune cell infiltration^44^. Other recent work in pancreatic cancer has demonstrated that matrix metalloproteinase-cleaved collagen I can activate DDR1, driving NFkB-dependent stress adaptation and metabolic reprogramming^24^. Our spatial assays corroborated this concept in sinonasal carcinomas by showing enrichment of DDR1 and downstream effectors in regions enriched by cleaved collagen I. This situates DDR1 inhibition as a particularly promising therapeutic avenue, with the potential not only to impair tumor survival but also to remodel the stromal context and potentially facilitate immune infiltration. This therapeutic approach could be particularly compelling in IDH2mt sinonasal carcinomas, where high KIT expression coexists with activated DDR1 signaling, raising the possibility that broad, clinically approved TKIs such as dasatinib and imatinib - capable of targeting both kinases - could simultaneously direct multiple vulnerabilities and thus achieve heightened therapeutic efficacy.

The tumor microenvironment further adds to the complexity of these tumors. Our analysis of ligand-receptor interactions revealed active immunosuppressive communication via CD96 between MC1 and MC2 and T cells, although both IDH2mt and SMARCA4mt tumors appeared largely immunologically “cold”, as reflected by the overall low number of immune cells in our samples. CD96 belongs to the nectin and nectin-like family of immunoregulatory receptors and has been reported to act as a functional T-cell checkpoint, counterbalancing the costimulatory activity of CD226^45^. In the context of collagen remodeling and the known immunosuppressive role of DDR1 signaling, this could further support a mechanism of immune exclusion that parallels findings in pancreatic and other desmoplastic tumors, where stromal stiffness and DDR1 activation prevent effective T-cell infiltration. Interrupting this axis may therefore synergize with immunotherapies by converting immunologically “cold” tumors into responsive ones^46^.

A limitation of the present study is the lack of functional validation of the identified molecular dependencies. The rarity of IDH2- and SMARCA4-mutated sinonasal carcinomas and the absence of well-established cell lines or other functional models that recapitulate these molecular subgroups currently limit experimental interrogation of candidate vulnerabilities. To our knowledge, no dedicated in vivo models specifically representing these epigenetically and genetically defined sinonasal carcinoma classes are available. Future efforts should therefore focus on the establishment of such models to functionally validate the proposed signaling dependencies and to assess therapeutic vulnerabilities in a controlled setting.

Despite these limitations, the present work establishes a comprehensive single-cell resource for sinonasal carcinomas and highlights molecular vulnerabilities with immediate translational relevance. By demonstrating that IDH2mt and SMARCA4mt sinonasal carcinomas harbor distinct, targetable transcriptional states, we provide a framework for hypothesis-driven clinical exploration. This study exemplifies how multi-omic and spatially resolved profiling can identify expression-nominated therapeutic vulnerabilities, including KIT-, MET-, CDK4/6-, and DDR1-directed approaches, while emphasizing the need for functional and translational validation.

## Methods

### Cohort

Six FFPE tissue blocks from poorly or undifferentiated sinonasal carcinomas with known *IDH2* mutations as well as six *IDH2* wildtype sinonasal carcinomas with truncating or missense *SMARCA4* mutations were retrieved from the archives of the Institute of Pathology, Ludwig-Maximilians University Munich, Germany and the Department of Neuropathology at the Charité-Universitätsmedizin Berlin. Following one empty section for hematoxylin and eosin staining, three 25 µm sections were cut and immediately processed as described below.

The study has been conducted in accordance with the Declaration of Helsinki and in keeping with the rules of good clinical practice and according to the German laws and ethical standards. The study protocol was reviewed and approved by the Ethics Committee of LMU Munich (23-0832). The requirement for individual informed consent was waived by the Ethics Committee due to the retrospective, non-interventional nature of the study and the use of archived specimens obtained several years earlier, for which recontacting patients would have been impracticable and disproportionate.

### Nucleus extraction and single nucleus RNA sequencing

To extract nuclei, tissue samples were sectioned into three slices, each with a thickness of 25 µm. Tissue slices underwent deparaffinization and tissue dissociation according 10X Genomics Demonstrated Protocol (CG000632 RevA and CG000527 RevC), including utilizing the gentleMACS™ Octo Dissociator (Miltenyi Biotec, Germany) to obtain a single cell suspension which were then counted on MACSQuant10 flow cytometer (Miltenyi Biotec, Germany) stained with DAPI (1 µg/ml). In addition, nuclei enrichment was performed on the single cell suspension using Nuclei Isolation Kit (Miltenyti Biotec, Germany) according to the manufacturer’s guidelines. The nuclei enrichment was followed by a 1 ml PBS wash, filtered with a 30 µm separation filter, and then nuclei were counted for a final nuclei resuspension in 500 µl Tissue Resuspension Buffer as per the standard steps outlined by 10X Genomics.

For the hybridization process, additional minor modifications were made to the protocol: samples were incubated for 30 min at 42 °C, after which an additional 2 h incubation at 23 °C was introduced, followed by the 16 h incubation at 42 °C as described in the protocol. For GEM chip loading, the cell suspension volume was increased by 20% compared to the manufacturer’s standard recommendation.

Library concentrations were quantified using the 1X dsDNA High Sensitivity Assay Kit on a Qubit4 Fluorometer (ThermoFisher, USA). The size distribution of library fragments was assessed using DNA High Sensitivity reagents with an Agilent 1200 Bioanalyzer (Agilent Technologies, USA). Libraries were then diluted to a final concentration of 120pM using Illumina resuspension buffer and sequenced on a NovaSeq XP Series sequencing platform (Illumina, USA) using a 10B 200 cycles flow cell. Sequencing quality was ensured by adding 8% PhiX (Illumina, USA) to the final sequencing pool. FASTQ files were generated utilizing the onboard BCL Convert software.

### Preprocessing of snRNA-seq data

Raw reads were aligned and quantified by CellRanger, version 7.1.0 (10x Genomics). We used GRCh38 as a reference transcriptome. In our analysis, we utilized R version 4.4.1 (2024-06-14) along with the R packages: Seurat version 5.1.0 (including SeuratObject version 5.0.2 and SeuratDisk version 0.0.0.9021)^47,48^.

We applied the analytic workflow from Stuart et al.^49^, adjusting the thresholds for cell counts and percentage of mitochondrial genes separately for each individual sample (see Table 1). DoubletFinder (version 2.0.4), was used to predict and remove doublets. Gene expression values were normalized using the SCTransform function (version 2). For integration, we identified 3000 highly variable features across samples and used Harmony for batch correction and integration of twelve samples into a single Seurat object^50^.

**Table 1:** Cell filtering and quality control thresholds for 12 samples

Sample	Initial size, cell number	N feature thresholds (min; max)	% of mitochondrial genes threshold	Number of detected doublets	Final size, cell number
IDH2.1	3039	200; 7500	15	127	2738
IDH2.2	11623	200; 9000	10	474	10437
IDH2.3	4490	200; 9000	20	69	3973
IDH2.4	1328	200; 5000	20	48	1184
IDH2.5	9927	200; 9000	25	321	8772
IDH2.6	8983	200; 9000	10	300	8216
SMARCA4.1	3570	200; 9000	10	704	2591
SMARCA4.2	6848	200; 9000	10	120	6380
SMARCA4.3	2314	200; 7000	5	458	1619
SMARCA4.4	2120	200; 4000	6	17	1991
SMARCA4.5	10525	200; 10000	15	180	9928
SMARCA4.6	1923	200; 5000	10	47	1783

Principal component analysis (PCA) was employed to reduce dimensionality and uniform manifold approximation and projection (UMAP) analysis was used for visualization^51^. We determined nearest neighbor relationships and clusters using the original Louvain algorithm. The optimal number of principal components was based on the percentage of variance associated with each principal component and their cumulative contribution.

### Reference data sources and cell typing

For cell typing, we performed mapping of ten references: nine datasets and one additional dataset based on a mixture of Choi CA, Choi LP, Choi LN, and Choi NL, see Suppl. Table S5^52–57^. One of the reference datasets was based on the single-cell atlas of the airway epithelium^58^.

For cell type prediction, we applied multimodal reference mapping to all reference datasets separately^59^. Each cluster was then assigned a predicted cell type on the basis of the respective reference data set. With ten reference datasets, this resulted in ten cell type predictions per cluster. Here, the most frequent prediction was selected as the consensus annotation. Next, further subtyping within clusters was performed by comparing this integrated dataset to additional reference datasets and marker genes^14,60^. After adjusting the clusters during cell typing, we finally identified 16 non-malignant and one malignant cluster. The annotation pipeline is illustrated in Suppl. Fig. S1.

### Copy number variations and identification of tumor cells

Tumor cells were identified by inferring copy number variants from snRNA-data using the inferCNV package version 1.20.0^61^. For comparative analysis, copy number profiles from bulk DNA methylation analysis were available for all samples. Cells exhibiting copy number alteration (CNA) profiles characteristic of known genomic alterations in IDH2mt and SMARCA4mt tumors were classified as malignant.

For comparative analysis across the 12 snRNA-seq samples, we generated a single plot that included all samples. To overcome the limitations of visualizations within the inferCNV package, which imposes restrictions on the maximum cell count for a single plot, we downsampled all samples to match the size of the smallest sample containing 338 cells (see Fig. 1d).

### Cell-cycle scoring and regression and intratumoral analysis

Cell-cycle scoring and regression were employed to minimize the influence of cell-cycle effects on downstream analysis of malignant clusters and tumor microenvironment^62,63^. We subsetted the malignant cells and divided them into initial samples. The cell-cycle phases of malignant cells were determined by scoring genes specific to the S and G2/M phases using curated marker sets available in Seurat’s integrated pipeline^48,64^. We incorporated these scores into the SCTransform normalization process to regress out the impact of cell-cycle variability, alongside mitochondrial content. As for the whole dataset, we constructed a shared nearest neighbor graph using Harmony and identified four subpopulations of malignant cells. By reducing this confounding factor, the resulting PCA and Harmony integration better reflected biologically meaningful variation, enabling more accurate clustering and visualization. Finally, clusters were visualized using UMAP and the effect of cell-cycle regression was validated through feature plots and ridge plots of key cell-cycle genes, ensuring the robustness of the dataset for further functional and comparative analyses. For differential gene expression analysis, we compared each of the four malignant clusters against the combined set of the other three clusters. We used the Wilcoxon-Mann-Whitney test and considered genes differentially expressed if they were detected in at least 20% of cells in either group and had a log2 fold change of at least 0.25. We applied the Benjamini-Hochberg (BH) multiple correction method with a padj < 0.001.

After cell typing and intratumoral heterogeneity analysis we obtained a Seurat object that included 16 non-malignant and four malignant clusters (MC1-MC4).

### Gene set enrichment analysis

We performed gene set enrichment analysis (GSEA) and identified significant biological pathways specific for MC1-MC4. The package msigdbr (version 10.0.1) was used to extract 1615 Reactome and 50 Hallmark gene sets from the molecular signature database (MSigDB)^65–67^. The package fgsea (version 1.30.0) provided functions for the gene set enrichment analysis^65–67^. Differentially expressed genes (DEGs) ranked by the average log2 fold change were used as input for GSEA. GSEA was performed on gene sets of size between 15 and 500 genes. We adjusted *p*-values using the BH multiple correction method and considered gene sets significant at padj < 0.05.

### Neuroendocrine differentiation and POU2F3 transcriptional program

We quantified neuroendocrine (NE) differentiation scores using mean expression of neuroendocrine markers synaptophysin (*SYP*), chromogranin A (*CHGA*), *CD56/NCAM1*, and *INSM1*, as mentioned by Febres-Aldana et al.^21^. The activation of a POU2F3-driven transcriptional program was evaluated using a combined gene set comprising seven canonical POU2F3/tuft cell markers *POU2F3, TRPM5, SOX9, GFI1B, CHAT, ASCL2, AVIL* described by Huang et al., and the seven GSEA leading-edge genes *POU2F3, PIRT, FOXI1, HEPACAM2, KIT, BCL2, ARHGEF2* reported by Febres-Aldana et al.^21,68^. We applied the AddModule function of the Seurat package to compute program activity scores. We used the Wilcoxon-Mann-Whitney test to compare POU2F3 program scores in IDH2mt samples with scores in SMARCA4mt samples and adjusted *p*-values using the BH multiple correction method and considered the results significant at padj < 0.05.

### Transcriptional metaprograms

Non-negative matrix factorization (NMF) was performed independently on the SCT-normalized assay of each sample using the GeneNMF package (version 0.9.2)^69^. For each sample NMF ran across a range of factorization ranks (k = 4–9), including genes expressed in at least 5% of cells. Sample-specific NMF programs were integrated into consensus transcriptional metaprograms using cosine similarly. A total of 10 metaprograms (MPs) were derived, and low-quality or redundant metaprograms were excluded based on similarity metrics and total number of genes per metaprogram, resulting in a final set of five metaprograms retained for downstream analyses.

### Pathway and transcription factors activity inference

The decoupleR algorithm (R package decoupleR version 2.10.0) was used to estimate the activity states of 14 signalling pathways involved in tumorigenesis (MAPK, VEGF, JAK-STAT, Androgen, TNFa, EGFR, Estrogen, PI3K, WNT, TGF-β, Trail, p53, Hypoxia, NFkB)^47^. Pathway activities were inferred using PROGENy networks, which incorporate curated pathways and their target genes along with weighted interactions^70^. We used the human-specific weights and selected the top 500 most responsive genes ranked by *p*-value to fit the multivariate linear model.

For transcription factor (TF) activity estimation, we used CollecTRI networks^71^. The CollecTRI-derived regulons encompass signed TF-target gene interactions, aggregated from 12 diverse resources. We used databases GeneCards and PathCards to validate our findings^22,72^. We downloaded the human version of interactions and applied the univariate linear model method, setting a minimum threshold of five targets per source.

### Exploration of target landscape of clinical kinase drugs

The targets of clinical kinase inhibitors were retrieved from Klaeger et al. using an affinity cut-off of 1 µM to only consider high-affinity binding inhibitors^73^. We quantified kinase expression levels both across four malignant cell clusters and two groups IDH2mt-SMARCA4mt, proposing specific inhibitors as potential therapeutic options.

### Cell-cell communication analysis

To investigate intercellular communication networks in the tumor microenvironment, we applied the CellChat package (version 2.1.2) to a subset of snRNA-seq data containing MC1-MC4 and immune cell populations including CD4 and CD8 T cells, B cells, M1 and M2 macrophages, and neutrophils^74,75^. After subsetting the data and assigning cell identities, we inferred ligand-receptor interactions based on the curated CellChatDB.human database including information from the KEGG database and primary literature^76^. We identified over-expressed genes per cell group and over-expressed ligand-receptor interactions. For the selected cell groups, 2806 highly variable ligand-receptor pairs were used for signaling inference. The method triMean was used for calculating the average gene expression per cell group.

### DNA sequencing

Two unstained tissue sections (10 µm each) were prepared for DNA isolation. Tumor-rich areas were selected by light microscopic evaluation of corresponding hematoxylin and eosin–stained sections. These regions were subsequently macrodissected with a sterile scalpel, ensuring a minimum tumor cell proportion of 70%. DNA extraction was carried out in a semi-automated manner using the Maxwell RSC Blood DNA Kit on the Maxwell RSC 16 instrument (Promega, Germany) according to an optimized workflow. DNA concentrations were measured with a Qubit fluorometer (Thermo Fisher Scientific, USA).

Targeted DNA sequencing was performed using an expanded Archer VariantPlex Lung Focus Panel (IDT, USA), covering the following genes: *AKT1, ALK, BRAF, CTNNB1, CUL3, EGFR, ERBB2, ESR1, FGFR1, FGFR2, FGFR3, FGFR4, HRAS, IDH1, IDH2, KEAP1, KRAS, MEK1, MET, NFE2L2, NRAS, NTRK1, NTRK2, NTRK3, PIK3CA, PTEN, RB1, RET, ROS1, SMARCA4, STK11*, and *TP53*. Library preparation was carried out according to the manufacturer’s instructions. Sequencing was conducted on an Illumina NovaSeq XP platform (Illumina, USA) using a 1.5B 300-cycle flow cell. Bioinformatic processing and variant calling were performed with Archer Suite software version 7.2 (IDT, USA). Only likely pathogenic or pathogenic mutations according to ClinVar annotation were reported.

### Immunohistochemistry

Immunohistochemical analyses were performed on an automated staining platform (VENTANA BenchMark XT, Ventana Medical Systems, USA). For MET detection, the SP44 monoclonal antibody was applied as previously described. Additional immunostainings included MET (SP44 monoclonal antibody; Ventana, USA; dilution 1:100), KIT (c-KIT polyclonal antibody; Agilent/Dako; dilution 1:400) and BRG1 (EPNCIR111A monoclonal antibody, Abcam, USA; dilution 1:100). Antibody incubation and signal detection were carried out according to the manufacturers’ protocols. A continuous MET and KIT H-score was calculated by multiplying staining intensity (0-3) by the percentage of positive tumor cells, yielding a range from 0 to 300.

### Fluorescence in situ hybridization

Fluorescence in situ hybridization (FISH) for MET was performed on 4 µm sections from formalin-fixed, paraffin-embedded (FFPE) tissue microarray (TMA) blocks according to the manufacturers’ instructions. Briefly, sections were deparaffinized, dehydrated, and incubated in pre-treatment solution (Dako, Denmark) for 10 min at 95–99 °C, followed by enzymatic digestion in pepsin solution for 3–6 min at 37 °C. After washing and dehydration, slides were air-dried and hybridized with a dual-color MET/CEP7 probe (Abbott Molecular, USA). Slides were sealed, denatured at 82 °C for 5 min in a humidified atmosphere, and incubated overnight at 45 °C for hybridization. Post-hybridization washes were performed according to the manufacturer’s protocol, and nuclei were counterstained with 4′,6-diamidino-2-phenylindole (DAPI).

FISH signals were evaluated in at least 50 non-overlapping tumor cell nuclei per case using a fluorescence microscope (Axio Imager Z1, Zeiss, Germany). MET status was assessed according to two established scoring systems. Using the Cappuzzo criteria, cases with a mean MET gene copy number (GCN) ≥ 5 signals per tumor cell were classified as MET FISH positive. According to the PathVysion criteria, cases showing a MET/CEP7 ratio ≥ 2 were considered MET FISH positive.

## Supplementary information

Supplementary Material

## Data Availability

The data supporting this study is available on Figshare (DOI: 10.6084/m9.figshare.27925980). The repository includes the Seurat object containing raw samples. The remaining Seurat object with cell type annotations and investigation layers can be obtained from the Seurat object with raw data when running the six R scripts. Due to file size limitations, reference datasets used for multimodal reference mapping can be provided upon reasonable request. The repository also includes empty structured folders supporting the organization of the six R scripts. The full analysis pipeline is provided as six R Markdown (.Rmd) files, which contain the scripts used for data import, processing, analysis, and figure generation. A separate R script is included with custom functions used throughout the pipeline. The repository also contains a matrix file with kinase targets used for the analysis. All code and documentation will be made publicly available at Figshare (DOI: 10.6084/m9.figshare.27925980).

## Code availability

The full analysis pipeline is provided as six R Markdown (.Rmd) files, which contain the scripts used for data import, processing, analysis, and figure generation. A separate R script is included with custom functions used throughout the pipeline. The repository also contains a matrix file with kinase targets used for the analysis^73^. All code and documentation is publicly available at Figshare (10.6084/m9.figshare.27925980).
