# Supplementary material for: Single nucleus RNA profiling reveals potential therapeutic vulnerabilities in sinonasal carcinomas
Source: NPJ Precis Oncol. 2026 Jul 7;10:260. doi: 10.1038/s41698-026-01597-6 (PMC13342519; doi:10.1038/s41698-026-01597-6)
Supplement: Supplementary file 1 — Supplementary Material [file 41698_2026_1597_MOESM1_ESM.pdf]

Supplementary Material

Supplementary Figures

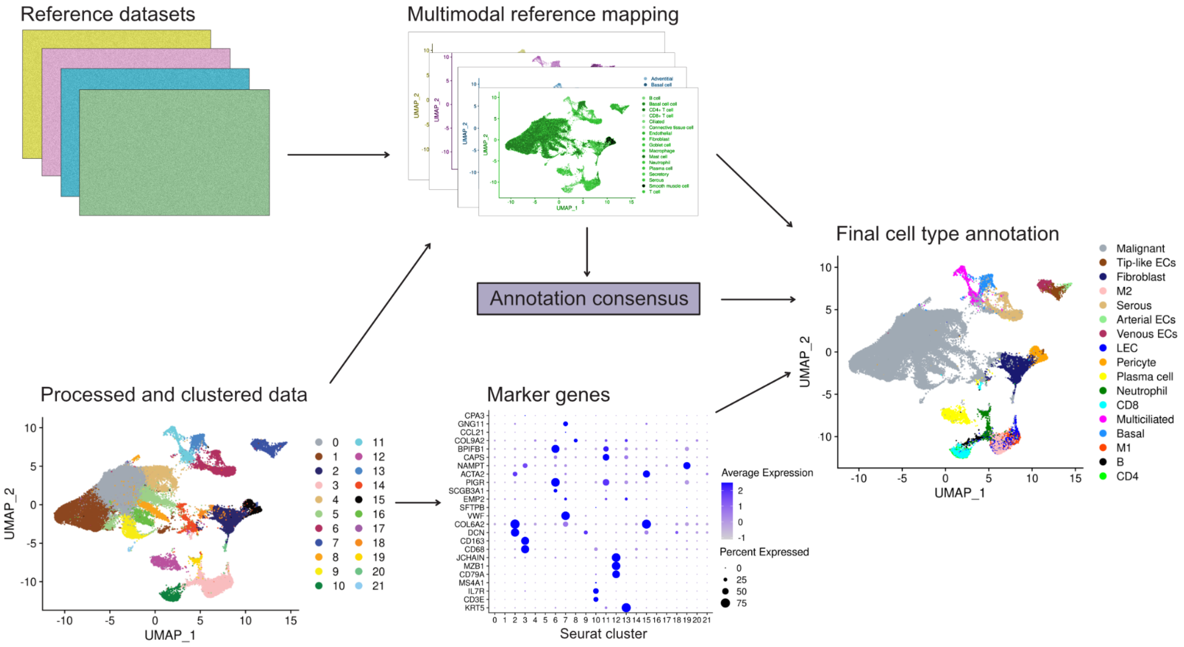

Supplementary Figure S1 Cell type annotation workflow. The workflow integrates reference mapping and canonical marker genes to achieve annotation consensus. Using multimodal reference mapping, we incorporated ten reference datasets to generate ten separate predictions for each cell. Additionally, we visualized canonical marker gene expression to facilitate cluster identification and refine the annotations. Annotation consensus was built from the prevailing cell type prediction for each cluster. By combining the results of reference mapping with annotation consensus and canonical marker genes, a robust annotation consensus was achieved. This led to the final cell type annotation. The resulting UMAP visualization contains 17 final cell types including malignant cells, various endothelial subtypes, fibroblasts, pericytes, M1 and M2 macrophages, neutrophils, CD8 T cells and CD4 T cells, B cells, basal cells, multiciliated cells and plasma cells, providing a comprehensive overview of the cell type landscape.

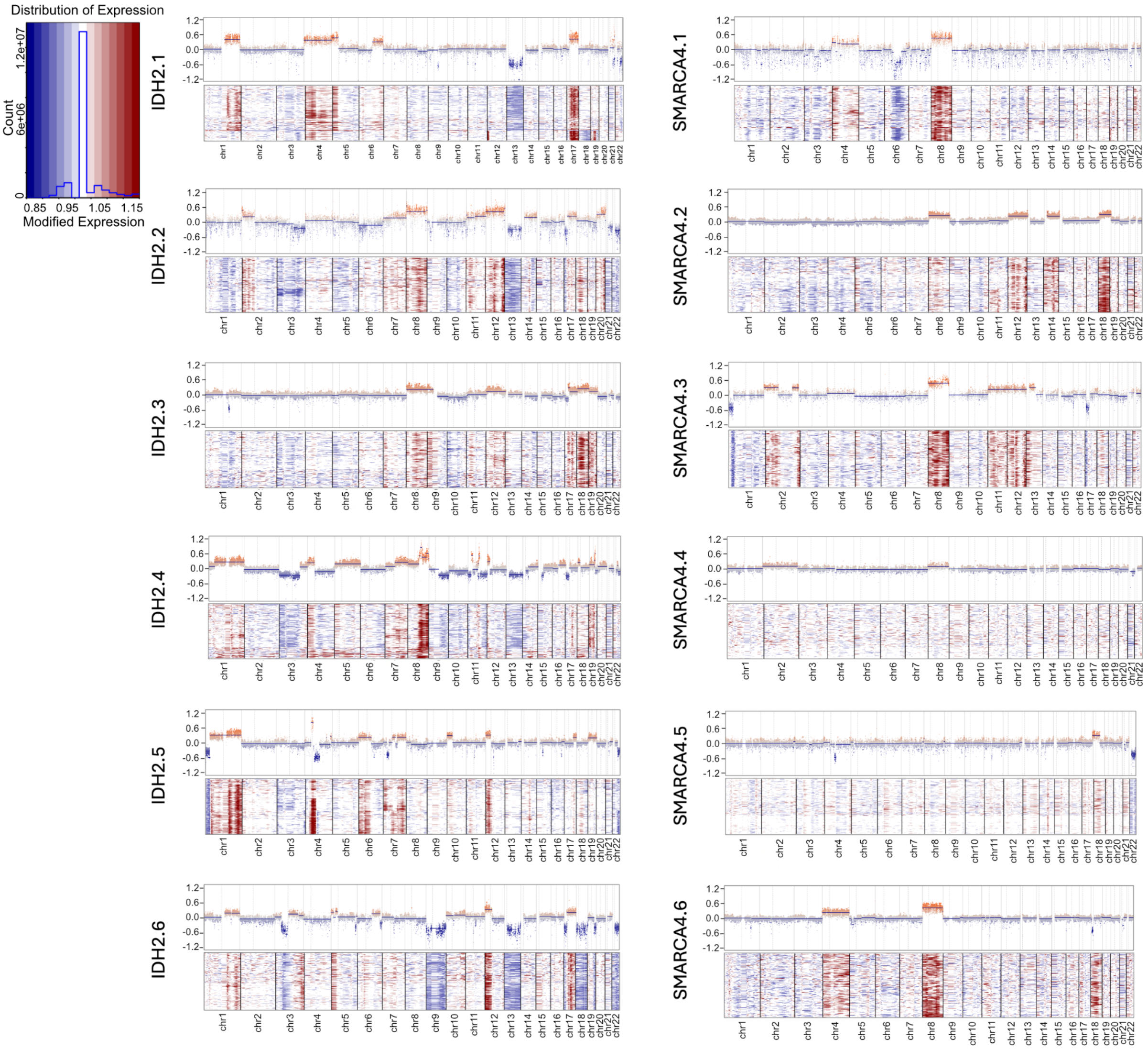

Supplementary Figure S2 Paired copy number variation (CNV) profiles of the entire genome of six *IDH2* mutated (IDH2mt) and six *SMARCA4* mutated (SMARCA4mt) samples. The top plots depict CNV insights inferred from snRNA-seq data, while the bottom plots represent CNV patterns from DNA methylation data. We observe a strong alignment between the CNV profiles derived from snRNA-seq and DNA methylation data.

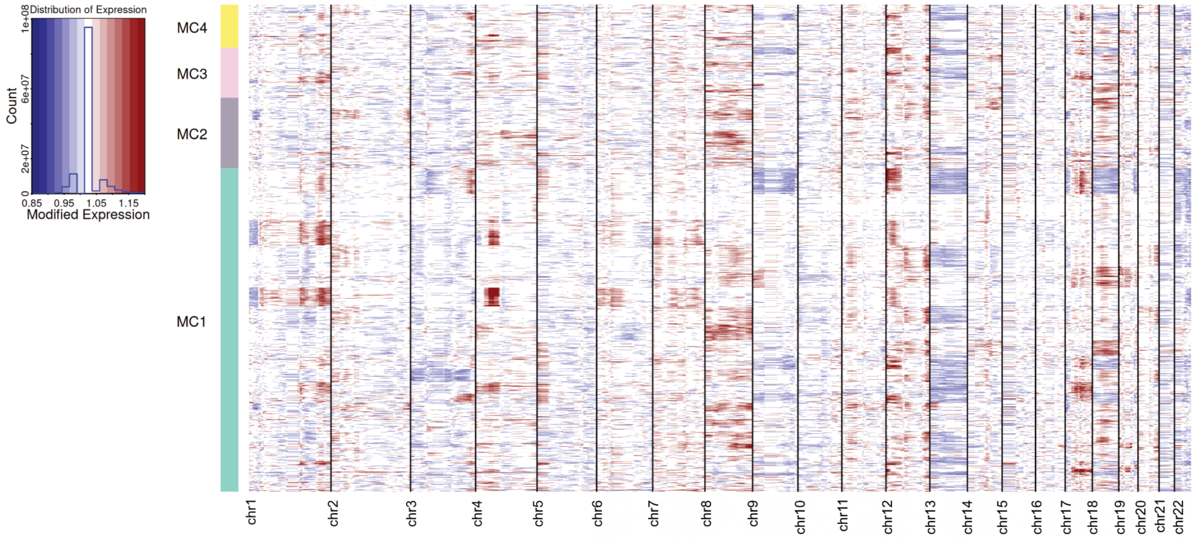

Supplementary Figure S3 Copy number variation (CNV) profiles inferred from the snRNA-seq data stratified by malignant clusters MC1-MC4. The malignant clusters were not characterized by cluster-specific CNV patterns.

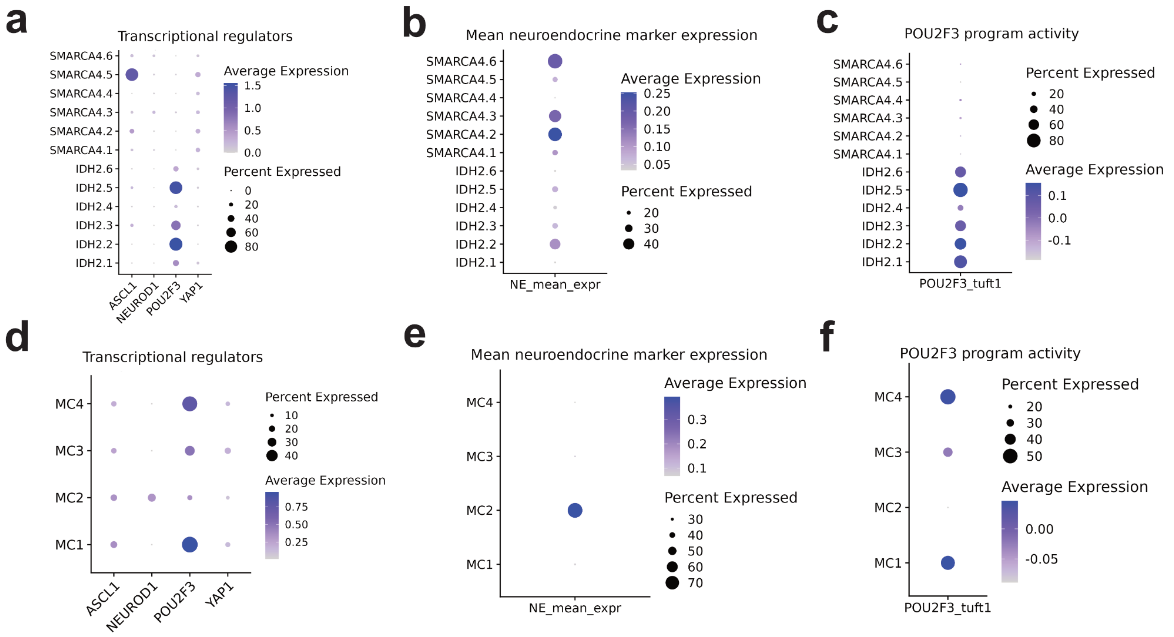

Supplementary Figure S4 Neuroendocrine differentiation (NE) and POU2F3-associated transcriptional program activity across tumors and malignant clusters (MC).
(a) Expression of transcriptional regulators across six *IDH2* mutated (IDH2mt) and six *SMARCA4* mutated (SMARCA4mt) tumors.
(b) The mean NE score across six IDH2mt and six SMARCA4mt samples. The mean NE score was determined by the mean expression of four neuroendocrine markers synaptophysin (SYP), chromogranin A (CHGA), CD56/NCAM1, and INSM1.
(c) The POU2F3 program activity of tumor cells for six IDH2mt and six SMARCA4mt samples. For assessing the POU2F3 program activity, the module score was computed from 13 genes associated with the POU2F3 transcriptional program. The mean sample POU2F3 module score is derived from the average of tumor cell scores.
(d) Expression of transcriptional regulators across malignant clusters MC1-MC4.
(e) The mean NE score across malignant clusters MC1-MC4.
(f) The POU2F3 program activity of tumor cells for four malignant clusters MC1-MC4.

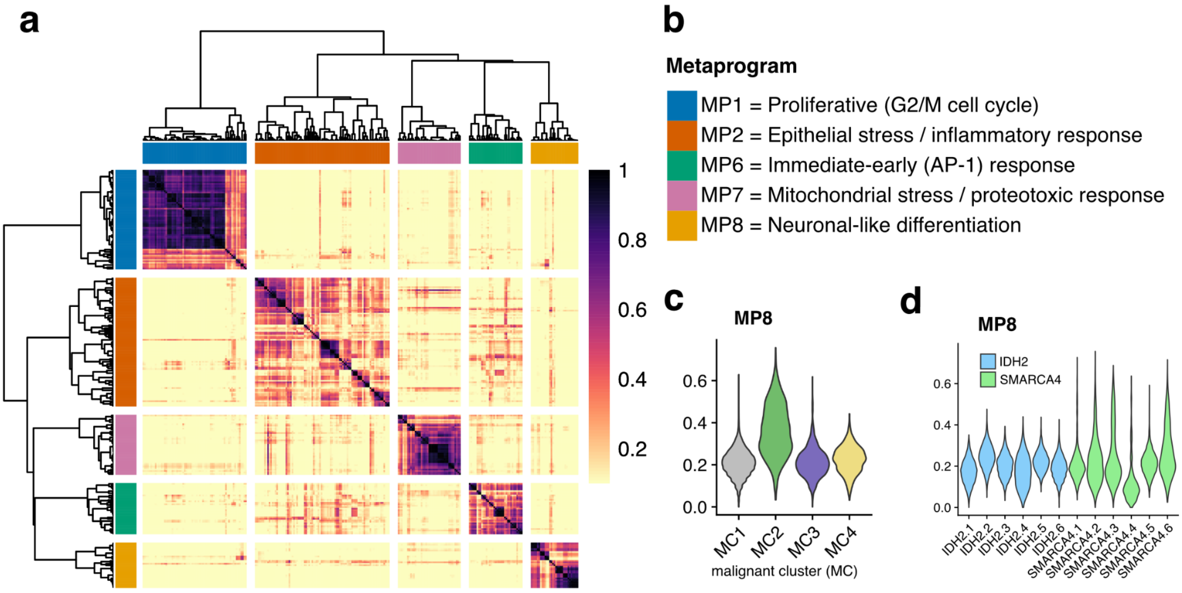

Supplementary Figure S5 Non-negative matrix factorization (NMF)-based identification of five metaprograms (MPs) in the tumor cell fraction.

(a) Correlation heatmap displaying the MPs.

(b) Summary of metaprogram labels.

(c) Fraction of MP8 stratified by malignant cluster (MC1-MC4).

(d) Fraction of MP8 stratified by tumor sample and color-coded by subgroup IDH2mt vs. SMARCA4mt.

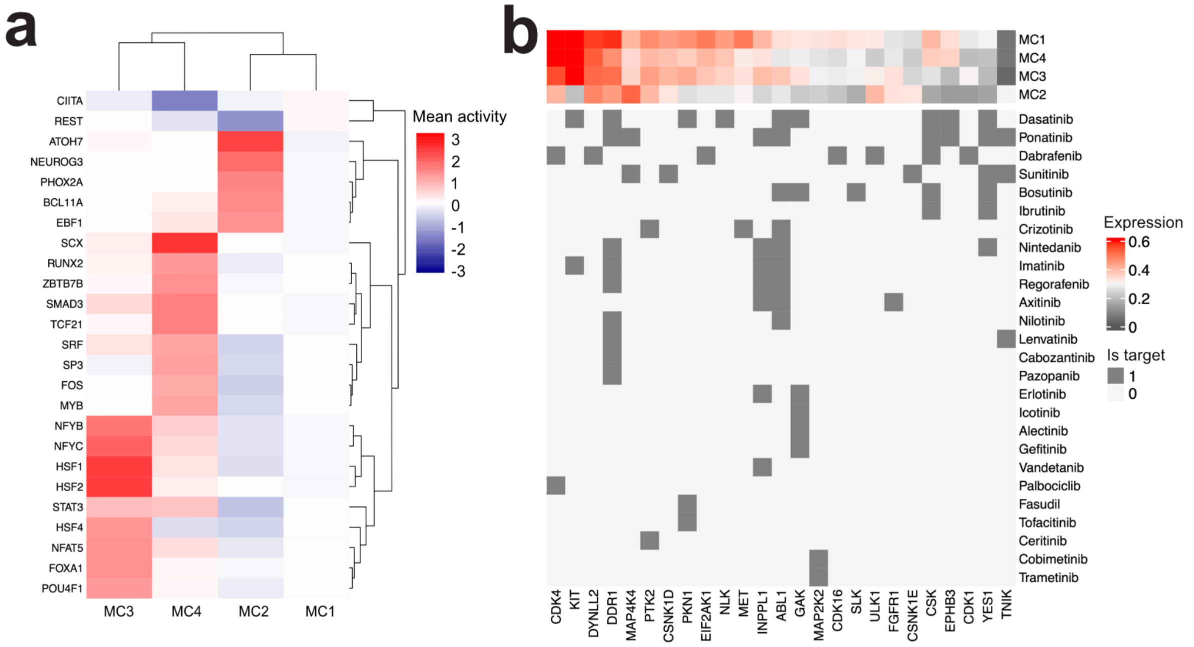

Supplementary Figure S6 Transcription factor (TF) activity and kinase target expression across malignant clusters (MCs).

(a) Transcription factor activity estimation across MC1-MC4. The heatmap shows TF activity levels estimated with decoupler. MC1 exhibits no detectable TF activity. MC2 demonstrates high activity of TFs ATOH7, NEUROG3, PHOX2A, BCL11A, and EBF1 suggesting its involvement in nervous system development. MC3 is characterized by elevated activity of NFYB, NFYC, HSF1, and HSF2, reflecting its role in cellular stress responses. MC4 shows strong activity of SCX, RUNX2, ZBTB7B, SMAD3, and TCF21, supporting its association with epithelial-mesenchymal transition (EMT) pathways and TGF-β-driven processes linked to tumor invasion and metastasis.

(b) Expression of clinical kinase drug targets and their corresponding inhibitors across MC1-MC4 adjusts our findings from the *IDH2* mutated (IDH2mt) and *SMARCA4* mutated (SMARCA4mt) groupings. CDK4 and DDR1 are broadly expressed across all clusters. KIT shows high expression in clusters MC1, MC3, and MC4, reflecting the high content of nuclei from IDH2mt samples in these clusters.

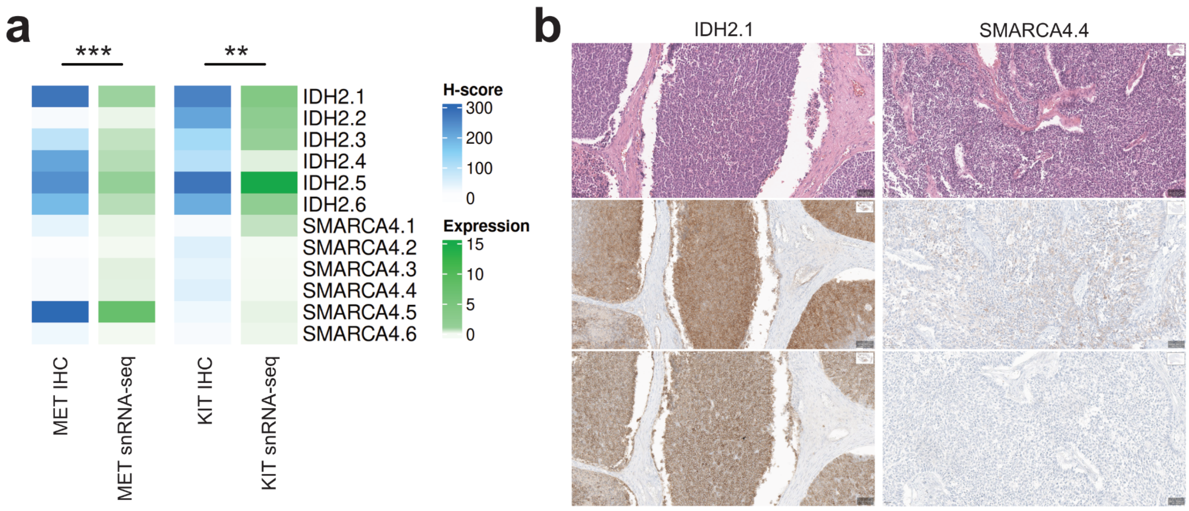

Supplementary Figure S7 Correlation of MET and KIT mRNA expression level with protein expression level in *IDH2* mutated (IDH2mt) IDH2mt and *SMARCA4* mutated (SMARCA4mt) tumors.

(a) Heatmap representation of MET and KIT expression levels measured by immunohistochemistry (H-score) and snRNA-seq across the full cohort. A strong concordance between transcript abundance and protein expression was observed for both MET (Spearman $\rho = 0.87$, $p < 0.001$) and KIT ($\rho = 0.75$, $p < 0.01$), demonstrating robust mRNA-protein correlation.

(b) Representative hematoxylin and eosin and immunohistochemical stainings illustrating concordant high and low expression patterns. IDH2.1 shows high KIT and MET expression at both mRNA and protein levels, whereas SMARCA4.4 exhibits low transcript levels and correspondingly weak immunohistochemical staining.

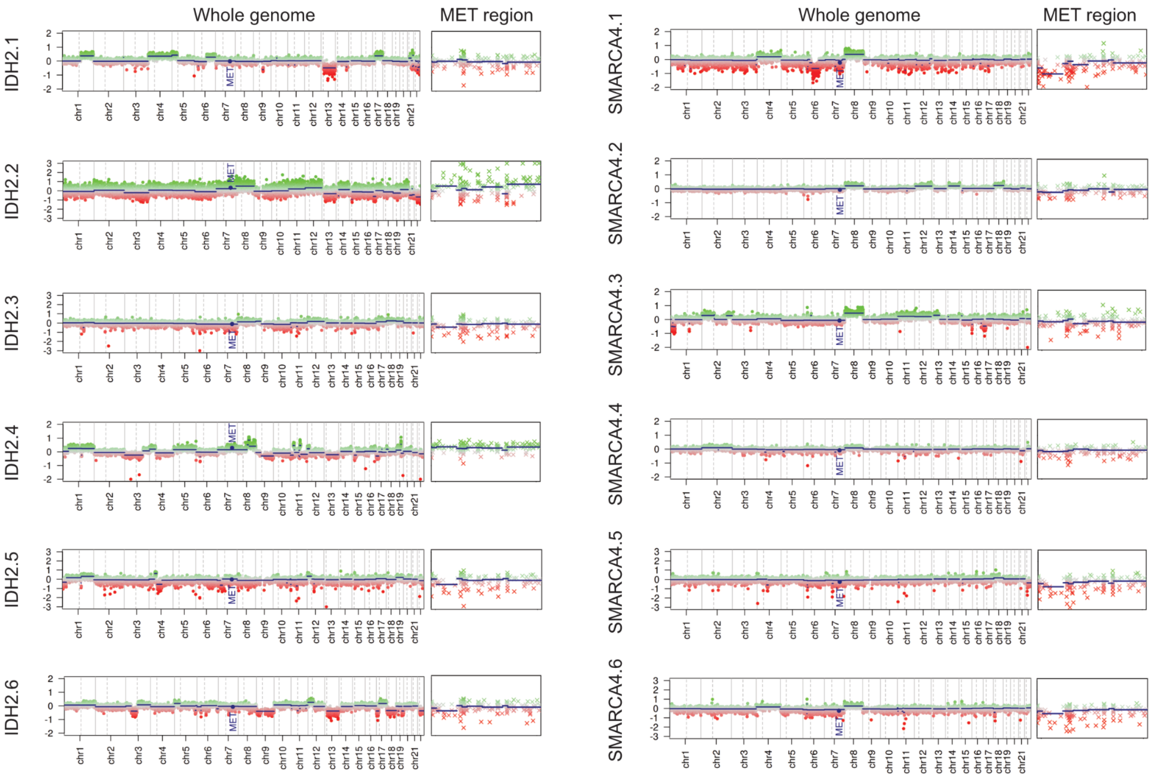

Supplementary Figure S8 Copy number variation (CNV) profiles of six *IDH2* mutated (IDH2mt) and six *SMARCA4* mutated (SMARCA4mt) samples inferred from DNA methylation data. The left plots represent CNV patterns of the entire genome, while the right plots depict CNV patterns of the MET region.

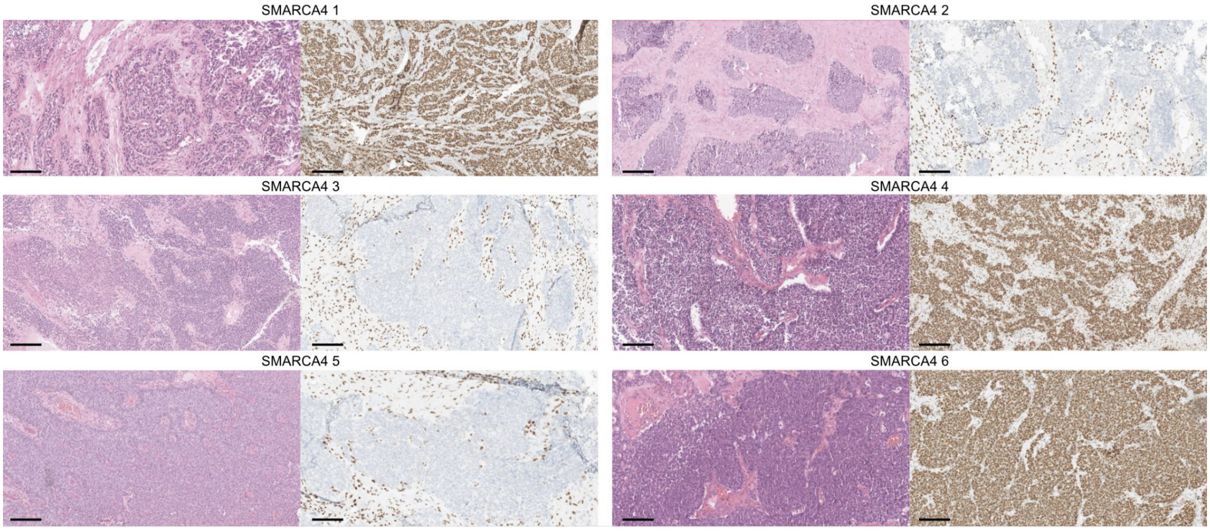

Supplementary Figure S9 Representative tumor hematoxylin and eosin and BRG1 immunohistochemistry images of six *SMARCA4* mutated samples. Tumors carrying truncating SMARCA4 mutations exhibit complete absence of nuclear BRG1 staining, consistent with loss of functional protein expression.

Supplementary Tables

ID	Nuclei count	Median reads per nucleus	Conf. mapped reads	FFPE creation
IDH2.1	3,039	9,271	66.55%	2017
IDH2.2	11,623	15,508	91.14%	2011
IDH2.3	4,490	13,526	81.82%	2017
IDH2.4	1,328	6,590	62.62%	2017
IDH2.5	9,927	21,968	92.75%	2022
IDH2.6	8,983	10,556	85.50%	2013
SMARCA4.1	3,570	13,110	79.34%	2015
SMARCA4.2	6,848	10,579	90.88%	2023
SMARCA4.3	2,314	7,623	64.44%	2005
SMARCA4.4	2,120	4,514	63.28%	2012
SMARCA4.5	10,525	14,534	93.95%	2020
SMARCA4.6	1,923	5,196	70.74%	2021

Supplementary Table S1 Sequencing metrics as retrieved by the CellRanger output report. Conf.= confidently, FFPE=formalin-fixed and paraffin-embedded.

	IDH2	SMARCA4	TP53	CTNNB1	PIK3CA	MET H-Score	KIT H-Score
**IDH2.1**	p.R172T	WT	WT	WT	WT	280	260
**IDH2.2**	p.R172T	WT	WT	WT	WT	20	210
**IDH2.3**	p.R172T	WT	p.E204X	WT	WT	90	120
**IDH2.4**	p.R172K	WT	WT	WT	WT	210	100
**IDH2.5**	p.R172M	WT	WT	WT	WT	240	280
**IDH2.6**	p.R172S	WT	WT	WT	WT	180	200
**SMARCA4.1**	WT	p.K785R	WT	p.T41I	WT	40	20
**SMARCA4.2**	WT	p.R1244Afs*47	WT	WT	p.H1047R	10	50
**SMARCA4.3**	WT	p.Q611*	WT	WT	WT	20	40
**SMARCA4.4**	WT	p.R1192C	WT	p.G34V	WT	20	50
**SMARCA4.5**	WT	p.Q306Rfs*12	WT	WT	p.E545K	300	30
**SMARCA4.6**	WT	p.G1232S	p.T125M	WT	WT	30	20

Supplementary Table S2 Molecular characterization of *IDH2* mutated (IDH2mt) IDH2mt and *SMARCA4* mutated (SMARCA4mt) tumors.

	MET/CEP7 ratio	Mean gene copy number
**IDH2.1**	1.0	2.0
**IDH2.2**	0.9	1.9
**IDH2.3**	1.0	2.1
**IDH2.4**	1.1	2.1
**IDH2.5**	1.2	2.2
**IDH2.6**	0.9	1.9
**SMARCA4.1**	1.0	2.0
**SMARCA4.2**	1.0	2.1
**SMARCA4.3**	0.9	1.9
**SMARCA4.4**	1.1	2.3
**SMARCA4.5**	1.0	2.1
**SMARCA4.6**	1.0	2.1

Supplementary Table S3 MET fluorescence in situ hybridization (FISH) analysis in *IDH2* mutated (IDH2mt) IDH2mt and *SMARCA4* mutated (SMARCA4mt) tumors. No evidence of MET amplification was detected in any analyzed case.

Target	Region	Mean spots/cell	p-Value
DDR1	cCol I positive	3.121	<0.001
	cCol I negative	0.006	
NRF1	cCol I positive	2.912	<0.001
	cCol I negative	0.002	
NFkB1	cCol I positive	1.012	<0.001
	cCol I negative	0.512	
SQSTM1	cCol I positive	2.151	<0.001
	cCol I negative	0.004	

Supplementary Table S4 Quantification of RNAscope results comparing the mean number of spots per cell between cleaved collagen I (cCol I) negative and positive tumor areas.

Dataset	Area	Sample count	Cell count	Cell types
PBMC	Peripheral blood	24	211,000	13 (57)
Tabula Sapiens L	Lung	3	35,682	40
Tabula Sapiens SG	Salivary gland	2	27,199	23
Tabula Sapiens T	Trachea	2	9,522	21
Airway epithelium	35 distinct locations, from the nose to the 12th division of the airway tree	10 (healthy)	77,969	9
Choi CA	Primary cancer tissue	20 (HNSCC)	18,838	9
Choi LP	Precancerous leukoplakia tissue	4 (HNSCC)	5,878	6
Choi LN	Metastatic tumors in the lymph nodes	4 (HNSCC)	7,747	9
Choi NL	Non-tumoral surrounding	9 (HNSCC)	15,189	9

Supplementary Table S5 Reference datasets. PBMC=dataset of peripheral blood mononuclear cells, L=lung, SG=salivary gland, T=trachea, HNSCC=head and neck squamous-cell carcinoma.
